# Enhanced variant neutralization through glycan masking of SARS-CoV-2 XBB1.5 RBD

**DOI:** 10.1080/22221751.2025.2502011

**Published:** 2025-05-06

**Authors:** Joey Olivier, Charlotte George, Chloe Qingzhou Huang, Sneha B. Sujit, Paul Tonks, Diego Cantoni, Joe Grove, Laura O’Reilly, Johannes Geiger, Christian Dohmen, Verena Mummert, Anne Rosalind Samuel, Christian Plank, Rebecca Kinsley, Nigel Temperton, Martina Pfranger, Ralf Wagner, Jonathan L. Heeney, Sneha Vishwanath, George W. Carnell

**Affiliations:** a Lab of Viral Zoonotics, Department of Veterinary Medicine, University of Cambridge, Cambridge, UK; b DIOSynVax Ltd, University of Cambridge, Cambridge, UK; c MRC-University of Glasgow Centre for Virus Research, University of Glasgow, Glasgow, UK; d Ethris GmbH, Planegg, Germany; e Viral Pseudotype Unit, Medway School of Pharmacy, University of Kent and University of Greenwich, Chatham, UK; f Institute of Medical Microbiology and Hygiene, University of Regensburg, Regensburg, Germany; g Institute of Clinical Microbiology and Hygiene, University Hospital Regensburg, Regensburg, Germany; h One Virology, Wolfson Centre for Global Virus Research, School of Veterinary Medicine and Science, University of Nottingham, Nottingham, UK

**Keywords:** Glycan masking, neutralising antibodies, next generation vaccines, SARS-CoV-2, receptor binding domain

## Abstract

SARS-CoV-2 continues to evolve antigenically under the immune pressure exerted by both natural infection and vaccination. As new variants emerge, we face the recurring challenge of updating vaccines at significant financial cost to maintain their efficacy. To address this, novel strategies are needed to enhance the breadth of protection offered by vaccines or, at a minimum, extend their effectiveness over time. One such strategy is antigen modification. In this study, we introduce a glycosylation site into a binding but non-neutralizing epitope within the SARS-CoV-2 XBB.1.5 receptor binding domain (RBD) to redirect the immune response towards more potent neutralizing epitopes. Immunization of mice with this modified antigen via the mRNA vaccine platform resulted in a dramatic increase in neutralizing antibodies compared to the wild-type XBB.1.5 RBD, showing superior protection against a range of SARS-CoV-2 Omicron variants, from BA.2 to JN.1. Our findings reinforce the power of the glycan masking approach, which in combination with the now well-established mRNA vaccine platform can contribute to broader and better vaccines.

## Main text

Since its emergence in August 2022, the XBB lineage of SARS-CoV-2 has driven multiple waves of SARS-CoV-2 infection worldwide. It has given rise to Variants of Interest (VOIs; XBB.1.5, XBB.1.16, and EG.5) and in July 2023, a new Omicron subvariant emerged – BA.2.86, which gave rise to the lineage JN.1 [1,2]. Despite carrying more than 30 mutations in its spike protein relative to its BA.2 variant, BA.2.86 was shown not to be more immune evasive than other circulating variants [1,2]. However, the danger posed by BA.2.86 lies in its distinct antigenicity, improved ACE2 binding affinity and high transmission worldwide which resulted in the emergence of JN.1 [3]. This allowed BA.2.86 to persist through population transmission and afforded it the opportunity to evolve a more immune evasive variant, JN.1. JN.1 carries a L455S mutation in its spike that is not present in BA.2.86 [4]. In December 2023, the WHO recommended XBB.1.5 as the antigen in monovalent mRNA vaccines as it was demonstrated that the vaccine induced broadly neutralizing and cellular immune responses against EG.5.1 and emerging XBB variants. In our study, we employed glycan masking to the receptor binding domain (RBD) of the SARS CoV-2 XBB.1.5 spike protein using mRNA-LNPs as delivery modality. By introducing the P521N modification to mask the site of CR3022 binding, a neutralizing epitope for SARS-CoV, but not for SARS-CoV-2, we aimed to enhance elicitation of neutralizing antibodies to SARS-CoV-2 variants by diverting the immune response to neutralizing epitopes.

**Figure 1.**
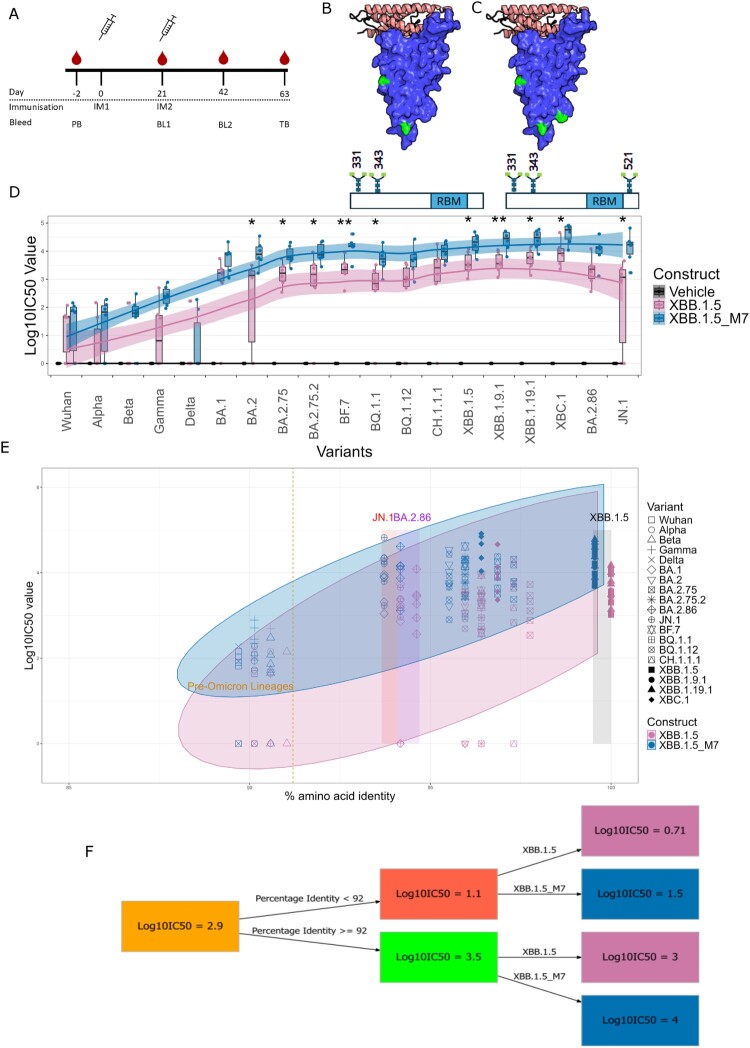
(A) Immunization schematic showing dates of immunizations and bleeds (PB, pre-bleed, BL1, bleed 1, BL2, bleed 2, TB, terminal bleed. IM1/IM2, immunization 1 or 2). (B, C) cartoon diagram of the SARS-CoV-2 XBB.1.5 RBD in complex with ACE-2 (pink). Glycosylation sites shown in green for XBB.1.5 (B) and the glycan masked construct (C). (D) Visualization of the Log_10_IC_50_ values obtained through pseudotype virus microneutralization assay with guinea pig serum after two immunizations with mRNA vaccines. The constructs against the different variants is given using box-and-whisker plots. A regression line (method = LOESS) with shaded confidence interval was added. The colours for the graph indicate the three constructs: Vehicle/naïve (black), XBB.1.5/WT (pink), XBB.1.5_M7 (blue). Mann–Whitney U-tests were performed to determine significance (*p* < 0.05 indicated by a single asterisk (*)). The graph was produced using the ggplot2 package in R. (E) A scatterplot of the Log_10_IC_50_ values and percentage identity between the variant RBDs and the different constructs is given. The different variants are indicated by distinct symbols as given by the legend. XBB.1.5/WT is given in pink, while XBB.1.5_M7 is given in blue. The different rectangles indicate the Log_10_IC_50_ values for both constructs for BA.2.86 (purple), JN.1 (red), and XBB.1.5 (black). A dashed dark yellow line indicates the separation between pre-Omicron variants and subsequent Omicron variants. The ellipse indicates the 95% confidence level. The graph was produced using the ggplot2 package in R. (F) A regression tree indicating the Log_10_IC_50_ value for the constructs when accounting for percentage identity. The regression tree model was produced using the R package rpart, while the plot was created using rpart.plot and DiagrammeR. The rules for the regression tree are as follows:

We have previously used the immune focusing approach of epitope masking in a proof-of-concept study in mice demonstrating that glycan modified RBD vaccines (delivered as DNA-prime MVA-boost regimen) generate potent binding and neutralizing antibody responses to the different SARS-CoV-2 lineages tested [5]. Here, we employ the same approach to an updated SARS-CoV-2 XBB.1.5 RBD and leverage the strengths of the mRNA platform developed by Ethris GmbH (http://www.ethris.com) in outbred guinea pigs to confirm enhanced immunogenicity of the glycan masked XBB.1.5 RBD vaccine (Figure 1A–C, Supplementary table 1, Supplementary Figures 1–3). We show once again that enhanced neutralizing antibody responses are generated when compared to the wild-type control across a panel of lentiviral pseudotypes bearing the native SARS-CoV-2 spike protein from its introduction into humans, subsequent variants and to the currently circulating lineage JN.1.1.

**Table:** 

Log_10_IC50_Value		cover
0.71	when Percentage_Identity < 92 & Construct is XBB.1.5	13%
1.5	when Percentage_Identity < 92 & Construct is XBB.1.5_M7	13%
3	when Percentage_Identity > = 92 & Construct is XBB.1.5	37%
4	when Percentage_Identity > = 92 & Construct is XBB.1.5_M7	37%

We immunized adult female Hartley guinea pigs twice with 15 µg LNP encapsulated mRNA encoding either the membrane tethered RBD of XBB.1.5 (hereby termed XBB.1.5_TM_RBD), the glycan masked RBD of XBB.1.5 (XBB.1.5_TM_M7_RBD), or vehicle controls, with a three-week interval. Blood serum was taken three weeks after prime and three weeks after boost, and terminal sera described here were collected six weeks after the second dose (Figure 1A). Sera were tested for neutralizing activity using lentiviral pseudotypes bearing the spike protein from a panel of SARS-CoV-2 variants of concern in pseudotype microneutralization assays (pMN), which have been shown to correlate with live virus assays [6]. The lentiviral pseudoviruses tested include Wu-Hu-1, Alpha, Beta, Gamma, Delta, BA.1, BA.2, BA.2.75, BA.2.75.2, BA.2.86, JN.1, XBB.1.5, XBB.1.9.1, XBB.1.19.1, BF.7, CH.1.1.1, XBC.1, BQ.1.1, BQ.1.12 [7].

When comparing XBB.1.5_TM_M7_RBD to XBB.1.5_TM_RBD, we detected a significant increase in neutralizing antibody titre (Log_10_IC_50_) in terminal sera against the grouped pseudotyped viruses for XBB.1.5_TM_M7_RBD (*p* < 0.0001, Mann–Whitney U test). Both XBB.1.5_TM_RBD and XBB.1.5_TM_M7_RBD produce a statistically significant difference in neutralizing antibody titre compared to the control group (*p* < 0.00001, Mann–Whitney U test). Interestingly, XBB.1.5_TM_M7_RBD elicited increased neutralization of immune evasive SARS-CoV-2 variants such as BA.2, BA.2.75, BA.2.75.2, BF.7, BQ.1.1, XBB.1.5, XBB.1.9.1, XBB.1.19.1, XBC.1, and JN.1 (*p* < 0.05; Mann–Whitney U-test) (Figure 1D). The largest fold change is seen with JN.1 (6.4) and BA.2 (5.7) (Supplementary Figure 4).

To ascertain whether XBB.1.5_TM_M7_RBD increased neutralization across the diversity of spike proteins captured in our pseudotype virus panel, we plotted neutralizing antibody titres against percentage amino acid identity relative to the Wu-Hu-1 RBD (Figure 1E). Following pairwise comparisons between XBB.1.5_TM_RBD, XBB.1.5_TM_M7_RBD and variant spike RBDs, we found that despite having only one amino acid difference from each other, XBB.1.5_TM_M7_RBD showed increased neutralizing antibody titre when compared with XBB.1.5. The neutralizing antibody titres for XBB.1.5_TM_M7_RBD are noticeably higher when the amino acid identity is lower when compared to XBB.1.5_TM_RBD, with ellipses in Figure 1(E) for both constructs indicating the 95% confidence level. The most parsimonious regression tree shows the effect of percentage amino acid identity and construct choice on neutralizing antibody titre (Figure 1F). When factoring in the percentage amino acid identity of the spike protein RBDs neutralized by the antisera, XBB.1.5_TM_M7_RBD elicited higher neutralizing antibody titres compared to the native XBB.1.5_TM_RBD sequence. Against spike protein RBDs where the amino acid identity to the vaccine is less than 92%, XBB.1.5_TM_M7_RBD elicited neutralizing antibody titres of ±1.5 compared to XBB.1.5_TM_RBD at ±0.71. In the case of a variant having an amino acid identity of more than or equal to 92%, XBB.1.5_TM_M7_RBD (±4.00) also outperformed XBB.1.5_TM_RBD (±3.00) (Figure 1F).

In summary, these results demonstrate significantly greater neutralizing antibody titre for XBB.1.5_TM_M7_RBD over the wild type XBB.1.5_TM_RBD, even with adjustment for amino acid similarity. Notably, this improvement in immunogenicity of XBB.1.5_TM_M7_RBD spanned four years of SARS-CoV-2 evolution, from Wu-Hu-1 to the recent JN.1 variant. This work provides further evidence for the strategy of immune focusing by masking a non-neutralizing epitope on the SARS-CoV-2 RBD.

## Discussion

Consistent with our first study, we demonstrate with a recent example that a glycan masking increases the neutralization capacity of SARS-CoV-2 RBD vaccine antigens. By introducing a single glycosylation site to mask the epitope associated with a binding but non-neutralizing antibody (CR3022) on the RBD of SARS-CoV-2 XBB.1.5, we have elicited enhanced neutralizing antibodies over that of the wild type antigen. This shows that glycan masking could be a powerful modification in antigen design, potentially future proofing against newly evolving SARS-CoV-2 variants.

When comparing the wild-type vaccine antigen (XBB.1.5_TM_RBD) against the modified vaccine antigen (XBB.1.5_TM_M7_RBD), XBB.1.5_TM_M7_RBD significantly enhanced neutralization capacity compared to the wild-type XBB.1.5 variant. This continued into the XBB-lineage, with both XBB.1.9.1 and XBB.1.19.1 spikes also being better neutralized by XBB.1.5_TM_M7_RBD.

JN.1 has less than 97% identity to XBB.1.5_TM_RBD and XBB.1.5_TM_M7_RBD, yet the XBB.1.5_TM_M7_RBD antigen still outperforms XBB.1.5_TM_RBD in terms of neutralization efficacy. These results are promising considering that a study has shown that JN.1 is more resistant to neutralization by both XBB.1.5 breakthrough infection and vaccine sera than BA.2.86 [4]. This may explain our results where BA.2.86 is neutralized by antisera generated by both XBB.1.5_TM_RBD and XBB.1.5_TM_M7_RBD vaccines with negligible differences, but that XBB.1.5_TM_M7_RBD neutralizes JN.1 significantly better than XBB.1.5_TM_RBD.

Antigenic cartography has shown that Omicron variants are antigenically distinct from the earlier VOCs [8]. Within the Omicron lineage, BQ.1 and XBB are not only antigenically distinct from each other, they are also antigenically distinct from early Omicron variants [8]. More recent antigenic maps have shown that BA.2.86 is distinct from D614G, BA.2, BA.5 and XBB.1.5, which suggested potential evasion of XBB-derived neutralizing antibodies [9]. BA.2.86’s descendant, JN.1, is antigenically distinct and distant from preceding variants [10]. It would be interesting to determine where XBB.1.5_TM_M7_RBD is positioned on these antigenic maps, and more importantly whether it can bridge the antigenic distance between relevant variants.

Hu *et al.* postulated that SARS-CoV-2 has evolved to have five serotypes (Ia, Ib, II–V) based on RBD antigenicity [11]. XBB.1.5_TM_M7_RBD significantly improves neutralization of pseudotyped viruses that fall in serotypes III (BA.2, BA.2.75), IV (BF.7, BQ.1.1), and V (XBB.1.5). It is likely that BA.2.86 and JN.1 would fall in a separate serotype (putative serotype VI). The BA.2 lineage (serotype III in Hu et al. [11]), gave rise to second-generation subvariants that are immune evasive and have driven multiple waves [12]. For example, not only did BA.2.75 exhibit increased immune evasion from vaccinated sera [13] it is also responsible for giving rise to extreme immune evasive variants such as CH.1.1 and XBB [14]. More specifically, XBB is a recombination of two BA.2 lineages, BJ.1 and BM.1.1.1 (derived from BA.2.75) [15]. It is thus reassuring to see that XBB.1.5_TM_M7_RBD significantly increases the neutralization of BA.2 and its descendants.

In conclusion, our results support antigenically enhanced constructs created by the addition of a glycan mask to improve and broaden their neutralization capacity. This strategy could serve as a valuable approach to update vaccine antigens, increasing the longevity of vaccine induced antibody responses and making them more resilient against emerging variants.

## Supplementary Material

Supplementary_v2-clean.docx
