# Supplementary material for: Enhanced variant neutralization through glycan masking of SARS-CoV-2 XBB1.5 RBD
Source: Emerg Microbes Infect. 2025 May 6;14(1):2502011. doi: 10.1080/22221751.2025.2502011 (PMC12093793; doi:10.1080/22221751.2025.2502011)
Supplement: Supplementary_v2-clean.docx [file TEMI_A_2502011_SM1055.docx]

### **Enhanced variant neutralisation through glycan masking of SARS-CoV-2 XBB1.5 RBD**

### **Authors**

Joey Olivier^1^, Charlotte George^1^, Chloe Qingzhou Huang^1^, Sneha B. Sujit^2^, Paul Tonks^1^, Diego Cantoni^3^, Joe Grove^3^, Laura O’Reilly^1^, Johannes Geiger^5^, Christian Dohmen^5^, Verena Mummert^5^, Anne Rosalind Samuel^5^, Christian Plank^5^, Rebecca Kinsley^2^, Nigel Temperton^4^, Martina Pfranger^6,7^, Ralf Wagner^6,7^, Jonathan L Heeney^1,2^, Sneha Vishwanath^1,2*^, George W. Carnell^1,2,8*^.

* Authors contributed equally

**Cambridge**

1. Lab of Viral Zoonotics, Department of Veterinary Medicine, University of Cambridge, Cambridge, UK

2. DIOSynVax Ltd, University of Cambridge, Cambridge, UK

3. MRC-University of Glasgow Centre for Virus Research, University of Glasgow, Scotland, UK

4. Viral Pseudotype Unit, Medway School of Pharmacy, The Universities of Kent and Greenwich at Medway, Chatham, UK

5. Ethris GmbH, Planegg, Germany

6. Institute of Medical Microbiology and Hygiene, University of Regensburg, Regensburg, Germany

7. Institute of Clinical Microbiology and Hygiene, University Hospital Regensburg, Regensburg, Germany

8. One Virology, Wolfson Centre for Global Virus Research, School of Veterinary Medicine and Science, University of Nottingham, United Kingdom

##

## **Supplementary Materials**

### **Materials and Methods**

#### **Design of vaccine constructs**

The sequence of the SARS-CoV-2 XBB.1.5 RBD was constructed as a consensus from XBB.1.5 strains obtained from NCBI genbank, and a glycosylation site was introduced at the CR3022 monoclonal antibody binding site to produce XBB1.5_M7. In-silico mutation of animo acids in the sequon N-X-T was carried out using the FoldX algorithm^1,2^. Briefly, two positions - 381 and 521 were chosen to introduce the glycosylation site in the XBB.1.5 RBD. The structure of the RBD was extracted from PDB id - 8V0R and first repaired using the Repair module of the FOLDx algorithm. The energy of the mutants was calculated using the BuildRepair module of FOLDx algorithm. Total 5 runs were performed for the BuildRepair module. We calculated the mutations with the least energy cost and used these in the design of XBB1.5_M7. Data shown in Supplementary Table 1

#### **Cells**

HEK293T/17 (ATCC: CRL-11268) cells were maintained and grown in Dulbecco’s MEM (DMEM) supplemented with 10% Fetal Bovine Serum (FBS, Merck) and 1% Penicillin/Streptomycin (Pen/Strep, Thermo Fisher) at 37°C and 5% CO2 in a humidified incubator.

#### **Pseudotype virus production**

Lentiviral pseudotypes were produced by transient transfection of HEK293T/17 cells with packaging plasmids p8.91^3,4^ and pCSFLW^5^ and different SARS-CoV-2 VOC spike-bearing expression plasmids using the Fugene-HD (Promega) transfection reagent^6,7^. 6-well plates pre-seeded with 2x10^5^ HEK293T/17 cells were transfected with 250 ng of p8.91, 375 ng of pCSFLW and 10-100 ng of pEVAC or pCAGGS spike bearing plasmids in 100 µl Opti-MEM for each well of a 6-well plate for transfection. Fugene-HD was added at 3 µl to 1 µg DNA ratio and incubated with DNA mixes for 15 minutes at R.T. Supernatants were harvested after 48h, passed through a 0.45 µm cellulose acetate filter (Merck Millipore), and titrated on HEK293T/17 cells transiently expressing human ACE-2 and TMPRSS2. Target HEK293T/17 cells were transfected 24h prior in a T75 flask with 2 µg pCAGGS-huACE-2 and 150 ng pCAGGS-TMPRSS2^8,9^.

#### **Flow cytometry binding analysis of vaccine antigen expression**

Naked vaccine mRNAs were transfected into HEK293T/17 cells using Lipoectamine MessengerMax transfection reagent (Thermo Fisher Scientific, cat LMRNA001). This assay was performed in triplicate or quadruplicate using 96-well plates 24h after 30,000 cells were seeded per well. 100 ng of mRNA was transfected per well with a 1:10 ratio of MessengerMax to Opti-MEM and incubated at R.T for 10 minutes. 24h after transfection, cells were detached with 0.25% Trypsin-EDTA and seeded into a 96-well V-bottom dilution plate for antibody staining. Briefly, cells were centrifuged at 300 x *g* for 2 minutes to pellet them, and washed with PBS -/-, 1% FBS. This was carried out twice, and cells resuspended in 50 µl PBS -/-, 1% FBS containing 2 µg/ml of CR3022 or S309, or 1:200 NIBSC serum 21/338 (<https://nibsc.org/documents/ifu/21-338.pdf>). Cells were incubated for 30 minutes at R.T, washed twice as previously and then incubated with secondary antibody (goat anti human AF647, Thermo Fisher Scientific) at 6 µg/ml in a volume of 50 µl for 30 minutes in the dark. Cells were then washed twice as previously, resuspended in 200 µl PBS -/-, 1% FBS including 7-AAD live/dead stain (Thermo Fisher, cat A1310) and read using the Attune NxT with autosampler (Thermo Fisher Scientific). Cells were gated for live singlets and then median fluorescence intensity was measured for bright far-red fluorescence. Untransfected and transfected controls for each transfected construct were tested with primary and/or secondary only controls to measure background fluorescence and used in the interpretation of the results.

#### **Animal Work**

Immunogenicity work was carried out in adult 8–10-week-old female Hartley Guinea pigs (Envigo/Inotiv). Two immunisations with a 21-day interval were performed by intramuscular injection. 100 µl of vehicle containing 15 µg lipid formulated mRNA was injected into each hind leg per immunisation. Blood was taken from the saphenous vein at day 21 and 42, three weeks after first and second immunisations. A final bleed was taken by cardiac puncture under terminal anaesthesia on day 63. Animal was carried out in accordance with U.K law through home office approved project license PP9157246. Ethics were approved by The Animal Welfare and Ethical Review Body and work carried out at University Biomedical Sciences facilities, University of Cambridge. Only data generated from the terminal serum (D63) is presented in this research letter.

#### **Pseudotype-based microneutralisation assays**

Pseudotype based microneutralisation assay was performed as described previously^10^. Briefly, serial dilutions of serum (terminal bleed only, collected on day 63, after second dose of mRNA vaccine) were incubated with SARS-CoV-2 spike bearing lentiviral pseudotypes for 1h at 37°C, 5% CO2 in 96-well white cell culture plates. 1.5x10^4 HEK293T/17 transiently expressing human ACE-2 and TMPRSS2 were then added per well and plates incubated for 48h at 37°C, 5% CO2 in a humidified incubator. Bright-Glo (Promega) was then added to each well and luminescence was read after a five-minute incubation period. Experimental data points were normalised to 100% and 0% neutralisation controls and non-linear regression analysis (Equation: log(inhibitor) vs. normalized response - Variable slope) performed to produce neutralisation curves and associated IC50 values. Internal standards consisting of a pool of sera from SARS-CoV-2 immunised animals as well as human international standards (NIBSC 21/338) were used to calibrate this assay.

#### **Pairwise comparison**

Jalview version 2.11.3.2^11^ was used to perform pairwise comparisons of the RBD sequences. Consensus sequences were used for the respective RBD sequences.

#### **Data and Statistical Analysis**

Neutralisation data was analysed and Log10IC50 values were determined using GraphPad Prism version 10.2.3 for Windows, GraphPad Software, Boston, Massachusetts USA ([www.graphpad.com](http://www.graphpad.com/)). R Statistical Software (v4.3.3; R Core Team 2024)^12^, using RStudio (Rstudio Team, 2024), was used to create graphs and perform statistical analyses. Mann-Whitney U-tests were used to determine the significance of Log10IC50 values between constructs, and False-Discovery Rate adjustment was used for all p-values. The effects of construct choice and percentage identity between variants and constructs on Log10IC50 values were examined using regression tree models estimated using the rpart package^13^.

## Supplementary figures

Supplementary Table 1: Differences in the energies of the vaccine antigen XBB.1.5_M7_TM_RBD with respect to XBB.1.5_TM_RBD. A total of 5 runs were carried out for the energy calculations presented. The standard deviation (SD), the mean total energy and mean of the components energies are reported.

| Position | SD | Total energy | Backbone Hbond | Sidechain Hbond | Van der Waals | Electrostatics |
| --- | --- | --- | --- | --- | --- | --- |
| 381 | 0.034509 | 1.17907 | -0.46374 | -0.46374 | -0.46374 | -0.75132 |
| 521 | 0.000905 | 0.673453 | -0.49545 | -0.49545 | -0.49545 | -0.01453 |


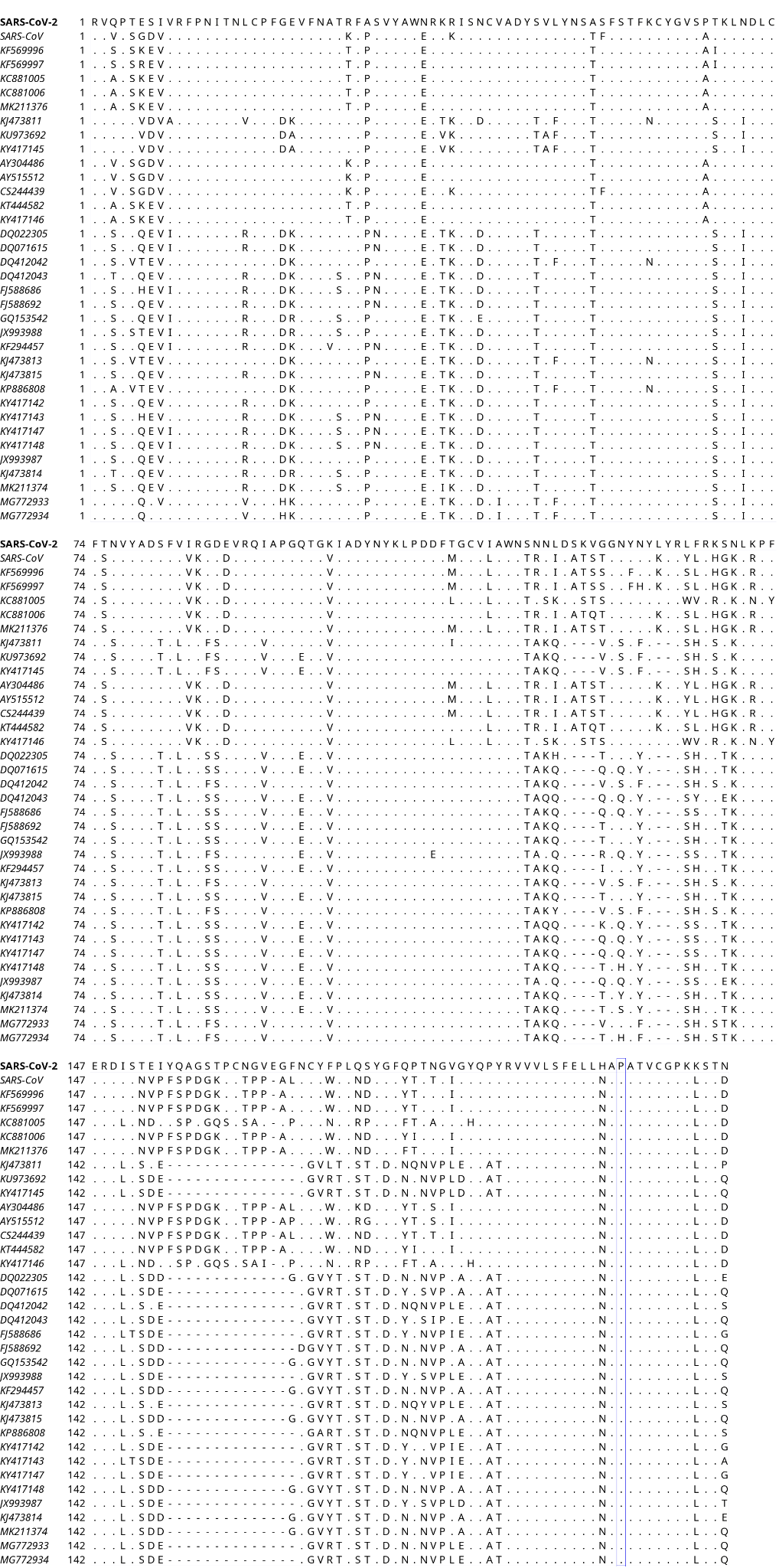


Supplementary figure 1.

Multiple sequence alignment of diverse Sarbecoviruses. The Wu-Hu-1 strain (NCBI ID: NC_045512.2) is used as reference and the conserved residues are represented as dots. The position 521 which was chosen for our vaccine modification to introduce a glycan, is boxed in blue, showing conservation of the residue despite the diversity of sequences shown.


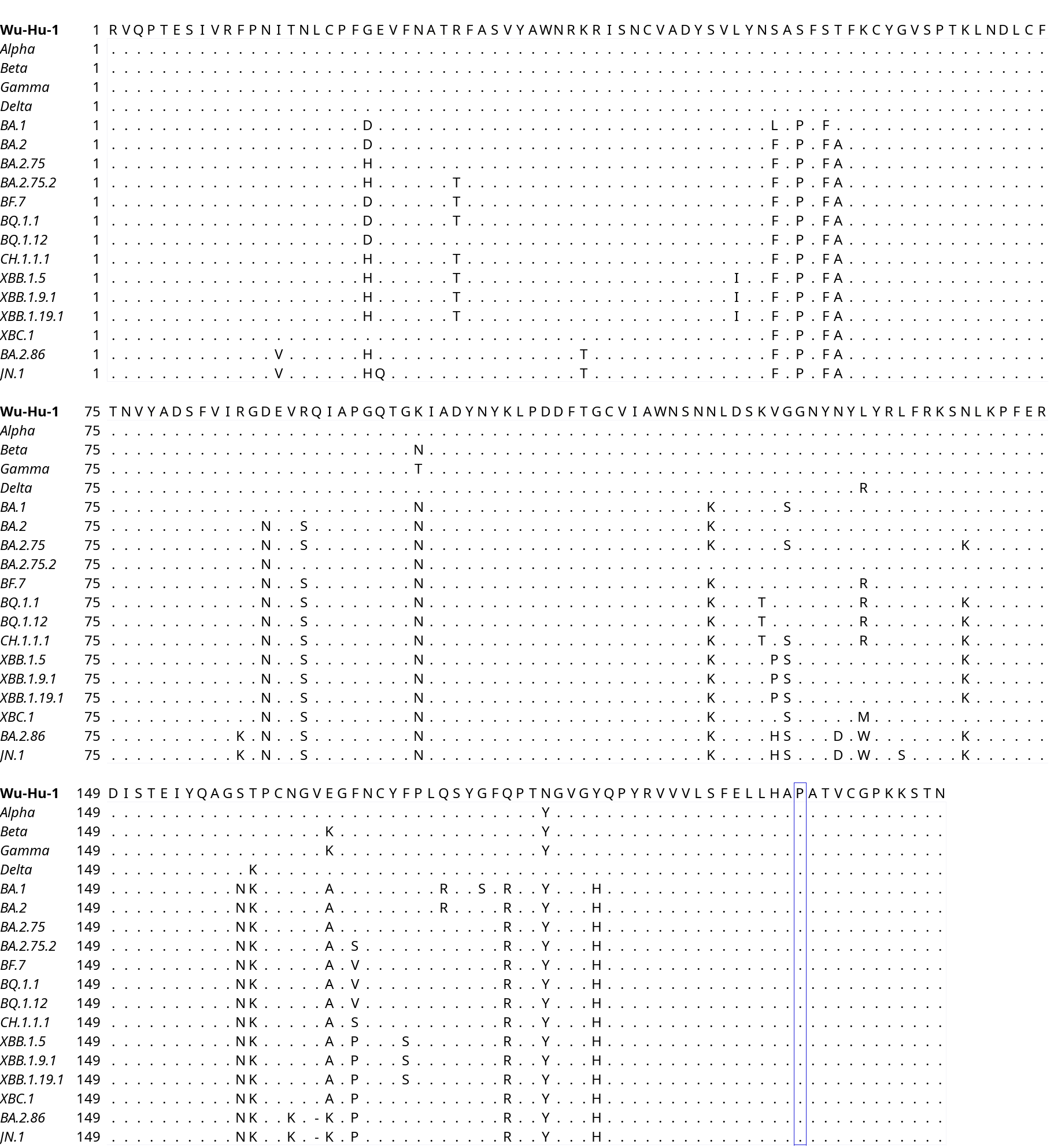


Supplementary figure 2.

Multiple sequence alignment of SARS-CoV-2 lineages. The Wu-Hu-1 strain (NCBI ID: NC_045512.2) is used as reference and the conserved residues are represented as dots. The position 521 which was chosen for our vaccine modification to introduce a glycan, is boxed in blue, showing full conservation across the lineages from 2019 to 2024..


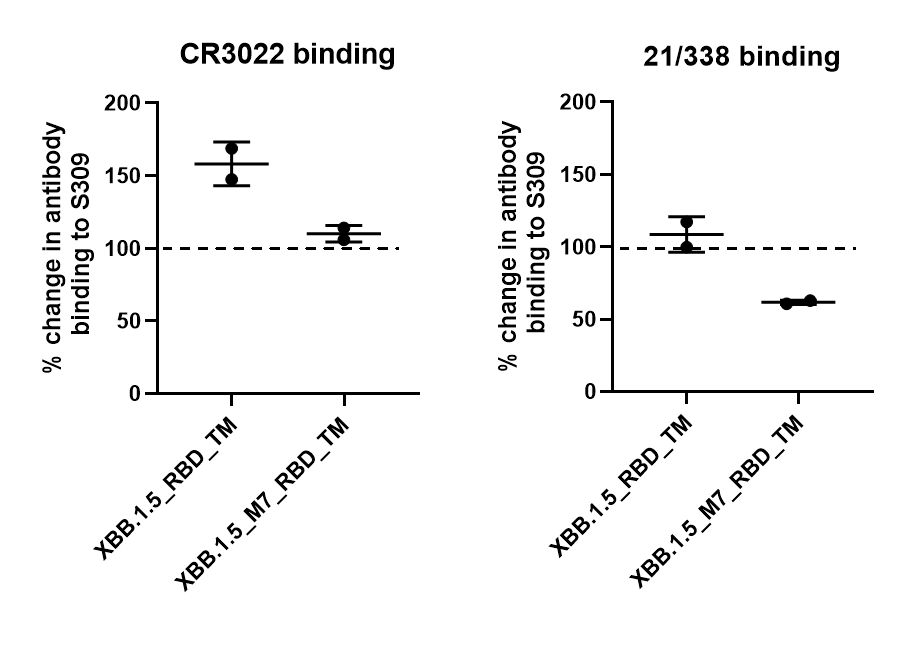


Supplementary figure 3.

Binding of antibody CR3022 and polyclonal antisera 21/338 to vaccine constructs XBB.1.5_RBD_TM and XBB.1.5_M7_RBD_TM. Values are normalised to S309 binding per construct (Geometric mean 8625 and 7940 MFI respectively) and represented by the dashed line at 100%. Binding of the antibody CR3022 is reduced for XBB.1.5_M7_RBD_TM as a result of the glycosylation site introduced to mask its binding epitope, whereas the binding of S309 is unchanged. The binding of polyclonal antisera 21/338 is also reduced for the XBB.1.5_M7_RBD_TM antigen as a result of the masked epitope.


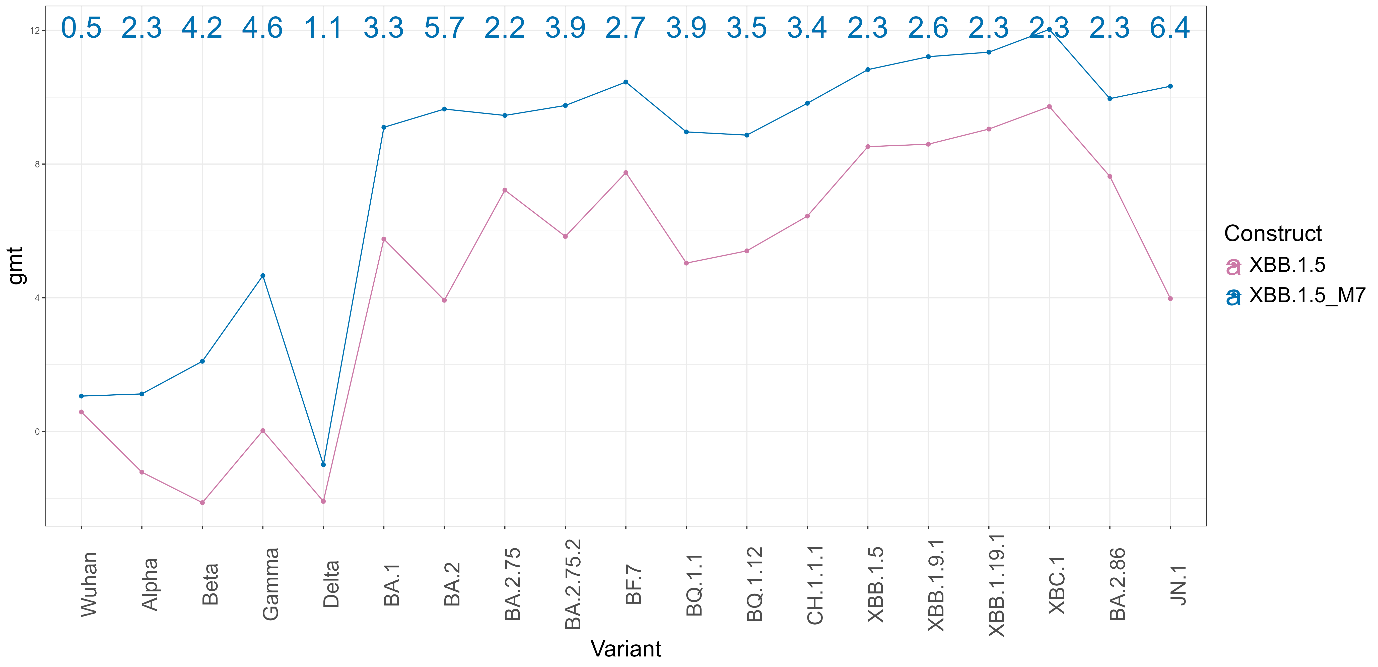
Supplementary figure 4.

Fold change in neutralising antibody titre elicited between XBB.1.5_RBD_TM and XBB.1.5_M7_RBD_TM antigens. The geometric mean titer was calculated using the titertools package^14^ in RStudio on the IC50 values (blue and pink lines), returning the log2 geometric mean titer (gmt). The fold change between XBB.1.5_RBD_TM sera and XBB.1.5_M7_RBD_TM sera was calculated for each variant and is displayed by the numbers in blue, top of the graph.

## **References**

1. Schymkowitz, J. *et al.* The FoldX web server: an online force field. *Nucleic Acids Res.* **33**, W382-388 (2005).

2. Kornfeld, R. & Kornfeld, S. Assembly of asparagine-linked oligosaccharides. *Annu. Rev. Biochem.* **54**, 631–664 (1985).

3. Zufferey, R., Nagy, D., Mandel, R. J., Naldini, L. & Trono, D. Multiply attenuated lentiviral vector achieves efficient gene delivery in vivo. *Nat. Biotechnol.* **15**, 871–875 (1997).

4. Naldini, L. *et al.* In Vivo Gene Delivery and Stable Transduction of Nondividing Cells by a Lentiviral Vector. *Science* **272**, 263–267 (1996).

5. Demaison, C. *et al.* High-Level Transduction and Gene Expression in Hematopoietic Repopulating Cells Using a Human Imunodeficiency Virus Type 1-Based Lentiviral Vector Containing an Internal Spleen Focus Forming Virus Promoter. *Hum. Gene Ther.* **13**, 803–813 (2002).

6. Sampson, A. T. *et al.* Coronavirus Pseudotypes for All Circulating Human Coronaviruses for Quantification of Cross-Neutralizing Antibody Responses. *Viruses* **13**, 1579 (2021).

7. Genova, C. *et al.* Production, Titration, Neutralisation, Storage and Lyophilisation of Severe Acute Respiratory Syndrome Coronavirus 2 (SARS-CoV-2) Lentiviral Pseudotypes. *BIO-Protoc.* **11**, (2021).

8. Hoffmann, M. *et al.* SARS-CoV-2 Cell Entry Depends on ACE2 and TMPRSS2 and Is Blocked by a Clinically Proven Protease Inhibitor. *Cell* **181**, 271-280.e8 (2020).

9. Bertram, S. *et al.* Influenza and SARS-Coronavirus Activating Proteases TMPRSS2 and HAT Are Expressed at Multiple Sites in Human Respiratory and Gastrointestinal Tracts. *PLoS ONE* **7**, e35876 (2012).

10. Carnell, G., Grehan, K., Ferrara, F., Molesti, E. & Temperton, N. An Optimized Method for the Production Using PEI, Titration and Neutralization of SARS-CoV Spike Luciferase Pseudotypes. *Bio-Protoc.* **7**, e2514 (2017).

11. Waterhouse, A. M., Procter, J. B., Martin, D. M. A., Clamp, M. & Barton, G. J. Jalview Version 2—a multiple sequence alignment editor and analysis workbench. *Bioinformatics* **25**, 1189–1191 (2009).

12. R Core Team. R: A Language and Environment for Statistical Computing. R Foundation for Statistical Computing (2024).

13. Terry Therneau, Beth Atkinson, Brian Ripley. rpart: Recursive Partitioning and Regression Trees. (2023).

14. Wilks SH (2023). titertools: A statistical toolkit for the analysis of censored titration data. R package version 0.0.0.9003.
